# Beyond greenspace: an ecological study of population general health and indicators of natural environment type and quality

**DOI:** 10.1186/s12942-015-0009-5

**Published:** 2015-04-30

**Authors:** Benedict W Wheeler, Rebecca Lovell, Sahran L Higgins, Mathew P White, Ian Alcock, Nicholas J Osborne, Kerryn Husk, Clive E Sabel, Michael H Depledge

**Affiliations:** European Centre for Environment and Human Health, University of Exeter Medical School, Truro Campus, Knowledge Spa, Royal Cornwall Hospital, Truro, Cornwall TR1 3HD UK; Department of Paediatrics, University of Melbourne, Flemington Road, Parkville Melbourne, Australia; NIHR CLAHRC South West Peninsula (PenCLAHRC), Plymouth University Peninsula Schools of Medicine and Dentistry, N32, ITTC Building, Tamar Science Park, Plymouth, PL6 8BX UK; School of Geographical Sciences, University of Bristol, University Road, Bristol, BS8 1SS UK

**Keywords:** Greenspace, Blue space, Nature, General health, Census, UK, Salutogenesis

## Abstract

**Background:**

Many studies suggest that exposure to natural environments (‘greenspace’) enhances human health and wellbeing. Benefits potentially arise via several mechanisms including stress reduction, opportunity and motivation for physical activity, and reduced air pollution exposure. However, the evidence is mixed and sometimes inconclusive. One explanation may be that “greenspace” is typically treated as a homogenous environment type. However, recent research has revealed that different types and qualities of natural environments may influence health and wellbeing to different extents.

**Methods:**

This ecological study explores this issue further using data on land cover type, bird species richness, water quality and protected or designated status to create small-area environmental indicators across Great Britain. Associations between these indicators and age/sex standardised prevalence of both good and bad health from the 2011 Census were assessed using linear regression models. Models were adjusted for indicators of socio-economic deprivation and rurality, and also investigated effect modification by these contextual characteristics.

**Results:**

Positive associations were observed between good health prevalence and the density of the greenspace types, “broadleaf woodland”, “arable and horticulture”, “improved grassland”, “saltwater” and “coastal”, after adjusting for potential confounders. Inverse associations with bad health prevalence were observed for the same greenspace types, with the exception of “saltwater”. Land cover diversity and density of protected/designated areas were also associated with good and bad health in the predicted manner. Bird species richness (an indicator of local biodiversity) was only associated with good health prevalence. Surface water quality, an indicator of general local environmental condition, was associated with good and bad health prevalence contrary to the manner expected, with poorer water quality associated with better population health. Effect modification by income deprivation and urban/rural status was observed for several of the indicators.

**Conclusions:**

The findings indicate that the type, quality and context of ‘greenspace’ should be considered in the assessment of relationships between greenspace and human health and wellbeing. Opportunities exist to further integrate approaches from ecosystem services and public health perspectives to maximise opportunities to inform policies for health and environmental improvement and protection.

## Background

### Natural environments and health and wellbeing

Whilst the relationships between natural environments (from urban greenspace to wilderness), human health and wellbeing have been explored over several decades, there has been a rapid growth in research in this field, and associated policy interest, in recent years. The potential salutogenic (‘health creating’ [1]) effects of natural environments are of interest in terms of tackling a wide variety of issues, such as obesity [2], mental health [3-5], mortality [6], perceived general health [7,8], specific morbidities including cardiovascular disease and musculoskeletal complaints [3], birthweight [9], and recovery from surgery [10]. Natural environments have also been proposed as a tool to help reduce socio-economic health inequalities [6,11]. Mechanisms proposed for these relationships include psychological processes of attention restoration [12] and stress reduction [13], opportunity/motivation for increased physical activity [14], reduced exposure to air pollution [15], immunological function associated with exposure to ‘healthy’ ecosystems [16] and opportunity for social contact [17]. Reviews indicate that the evidence base is diverse, of mixed (often low) quality, and is by no means conclusive [2,18-22]. The research exploring the various individual mechanisms for these relationships has also produced mixed findings and it is likely that any mechanisms *“intertwine”* [18] as opposed to each operating in isolation.

### Type and quality of natural environment

It is possible that the relatively simplistic consideration of natural environments in much of the earlier research may, to some extent, explain part of the inconsistency within the evidence base. This is plausible as different types and qualities of environment might afford different activities and promote variable psychological responses in a multitude of contexts. Typically, all natural environments have been considered generically and aggregated into a measure of so-called ‘greenspace’ without further qualification as to type or quality. Measures of the density of, or proximity to, these generic ‘green spaces’ are classified as such based on cartographical databases of land use (such as the English Generalised Land Use Database (GLUD) [4,6]) or classifications of remotely sensed land cover data, such as the European CORINE data [23]. Normalised Difference Vegetation Index (NDVI), derived from satellite imagery, has also been used as a single measure of neighbourhood greenness [9]. This homogenisation of ‘greenspace’ fails to consider the variation in its type and quality, and has been highlighted as a significant weakness in the evidence in reviews of the literature [18,20,24,25].

Studies that have considered type or quality indicate that more comprehensive consideration of natural environments is warranted. For instance, research from the UK suggests that different *types* of urban greenspace (using a broad typology e.g. ‘sports’/‘natural’) may promote physical activity to different extents [26]. Research in the Netherlands considered self-reported general health, symptom scores and mental health in relation to, proximal greenspace, divided into ‘urban green’, ‘agriculture’ and ‘forest and nature’ areas [27]. This supported positive health effects of ‘greenspace’ proximity overall and found some variation across the three types of ‘greenspace’. Evidence of the health and wellbeing effects of exposure to ‘blue space’ (coastal and inland water features/environments) is growing, and is suggestive of positive associations [5,11,28]. Swedish research found an association between women’s mental health, through physical activity mechanisms, and environment types classified as ‘serene’ and ‘spacious’, following a factor analysis of perceived environmental characteristics [29]. Analysis of data on visits to natural environments has made use of self-reported classification of environment type, and indicated different strengths of association with psychological wellbeing outcomes [30].

Additional research exists regarding *preference* for characteristics of natural spaces, and while extensive in its consideration of both type and quality, evidence is limited in terms of relationships with specific health outcomes [23,31-33]. However, some types of landscapes do appear to be appreciated to a greater degree than others. The most consistent differentiation in perception and preference is found between ‘wild’ and more ordered and managed landscape types [34]. Expressed preferences vary according to factors such as prior experience, living context, culture and demographics [12,32].

Various aspects of environmental quality have been considered in relation to greenspace usage, primarily focussed around social amenity and environmental incivility (such as litter). Generally these studies rely on primary audits of local environments, using a variety of tools, and have largely been focussed on supportiveness for physical activity [35,36]. A study of four Dutch cities considered perceived naturalness amongst other quality characteristics such as accessibility, maintenance, absence of litter and safety using streetscape/greenspace audits. In analyses with cross-sectional questionnaire data on self-reported general health, these quality measures demonstrated additional explanatory power beyond simpler greenspace quantity measures [37].

Finally, linkages between biodiversity (*ecological quality* or *state*) and human health and wellbeing have been explored, though efforts have largely focussed on potential sources of new medicines, food provision and infectious diseases [38]. A recent systematic review of the health promoting effects of biodiverse environments identified only 17 studies, and while the evidence was suggestive of a positive relationship, the findings were mixed and firm conclusions could not be drawn [22]. Biodiversity measures used in the research were broad ranging, including participant-perceived biodiversity, researcher visual assessment of complexity, objectively measured bird, butterfly and plant species richness, NDVI (as a presumed correlate of biodiversity), and protected area density. The few studies that objectively measured aspects of biodiversity suggest associations with psychological wellbeing outcomes, but inconsistently. For example, in two detailed investigations in a UK city, one found an association between measured bird and plant species richness and some measures of psychological wellbeing [39], whilst another found associations with lay perception of species richness, but not objectively measured richness [40].

### Aims & research questions

The Beyond Greenspace project aimed to develop small-area measures reflecting environmental greenspace type and quality, which could be combined with social survey datasets to investigate associations with health and wellbeing outcomes, in a manner comparable to previous greenspace research [4,7]. The key research question was: How are average levels of self-reported health in small geographical areas associated with the type and quality of different natural environments in those areas? A secondary question was: Do any observed associations between environmental measures and self-reported health differ according to urban/rural context and area socio-economic status?

## Methods

This study adopted a cross-sectional ecological approach using routinely available secondary datasets. Several small-area indicators of environmental type and quality were constructed, and were analysed alongside data on self-reported health from the 2011 Census.

### Geographical units

To be amenable to analysis with population socio-economic and health datasets, environmental data were allocated to Lower-layer Super Output Areas (LSOAs) for England and Wales, and Data Zones (DZs) for Scotland. These zones are a statistical census geography that have been used previously for greenspace/health research [7,11]. LSOAs and DZs are designed to include a population of approximately 1,500, with a minimum of 1,000 and maximum 3,000 [41]. Environmental indicators were constructed across Great Britain, but analyses with census health data were restricted to English LSOAs for reasons outlined below.

### Population general health

The 2011 UK census asked every individual the simple question “How is your health in general?” with 5 possible answers: Very good; Good; Fair; Bad; Very bad. Whilst this self-reported general health status assessment is simple and subjective, it is strongly associated with more complex dimensions of physical and psychological health, and objective measures such as mortality [42,43]. Data for LSOAs on population health by age and sex were used to calculate directly standardised prevalence of good/very good (hereafter referred to simply as ‘good’) and bad/very bad (‘bad’) health by LSOA. It is of interest to investigate both ‘good’ and ‘bad’ health since these are not necessarily just the inverse of one another and both have been used in relevant studies prior to this [7,11].

### Environmental indicators

Environmental indicators were selected on the basis of a balance of a) characteristics perceived or theorised to relate to health and wellbeing, or with relevant evidence from other literatures (such as that on environmental preferences described above); b) availability of national data at sufficient spatial resolution and for relevant time periods; and c) policy relevance (e.g. subject to regulation or potentially amenable to policy intervention). Inevitably, there is a compromise to be struck between data that are ideally desirable, and data that are available for the required geographical coverage, resolution and time period [44]. For environmental quality indicators, the hypothesis is not that the specific measure is necessarily directly affecting health and wellbeing, but that the underlying broad construct is indicative of a natural environment (characteristic) of a ‘type’ or ‘quality’ that may be beneficial.

### Land cover type and diversity

A key set of environmental indicators were those relating to land cover type. As indicated above, much of the literature has dichotomised built and green environments; however there is evidence to suggest different relationships with health and wellbeing outcomes following (presumed) exposure to different types of natural environment [30,45]. In order to classify ‘greenspace’ types in more detail, we made use of the UK Land Cover Map (LCM) for 2007 [46]. The LCM 2007 maps land cover in detail across the UK using satellite imagery combined with topographical data, classifying land parcels into one of 23 categories^a^, based on UK Biodiversity Action Plan Broad Habitats. Detailed LCM data are available for a 25 m resolution grid (raster). These data were attributed to LSOA/DZ boundaries using areal interpolation, to produce an area proportion measure of each land cover type for each LSOA/DZ.

The 23 land cover type proportions were subsequently aggregated to 10 LCM Aggregate Classes to enable meaningful analysis (urban/suburban environment plus nine natural land cover types). The 23 class version was used to calculate an index of land cover diversity for LSOA/DZ, using a Shannon diversity index formula accounting for the number of different types, and their relative proportions within the area (see equation 1) [47].

1 $$ \mathrm{S}\mathrm{D}\mathrm{I}=-{\displaystyle \sum_{k=1}^m}\left({P}_k\right) \log \left({P}_k\right) $$

SDI: Shannon Diversity Index; P_k_: proportion of local area classified as land cover type k; m: number of land cover types present within the LSOA/DZ.

### Biodiversity indicator

As described above, there is mixed evidence indicating whether objectively measured biodiversity may be associated with increased health and wellbeing, but there are indications that the relationship is positive [22]. Biodiversity can be conceived of in numerous ways [48], but in order to obtain an indicator meeting the needs of this project, we focused on obtaining national, georeferenced species richness data. Given that we required a spatially comprehensive dataset at sufficient resolution to be broadly applicable to LSOA/DZ boundaries, we obtained data from the British Trust for Ornithology/BirdWatch Ireland/Scottish Ornithologists' Club Bird Atlas 2007–11 [49]. The atlas is based on a systematic evaluation of bird species occurrence, an indicator of biodiversity [50,51], and a count of bird species richness (number of different species observed) was obtained for each cell of a 10 km grid. These data were again allocated to LSOA/DZ boundaries using areal interpolation.

### Generalised landscape quality indicator

As a general indicator of landscape quality, data on surface (primarily river) water quality were used, measured as part of the European Water Framework Directive by national environmental agencies. Since surface water flows through a catchment into rivers and other water bodies, the quality of the water in those bodies is reflective of the quality (and degradation) of the wider catchment, for example being impacted by agricultural chemical runoff and other activities in the surrounding landscape [52]. Data were available from the Environment Agency for England and Wales, for 7,055 water sampling locations with a high resolution (1 metre) grid reference and a valid ‘ecological status’ rating in 2011 [53]. These ratings indicate overall ecological status based on various biological, chemical and hydrological measures, and grade quality on a 5 point scale: High, Good, Moderate, Poor and Bad. The score for each sampling point was converted to an integer scale (with High = 2, Moderate = 0 and Bad = -2). A simple inverse distance weighted interpolation procedure (using a weight of 2 and 12 nearest neighbours) was carried out on these scores to estimate the spatial variation in ‘landscape quality’ for a 1 km square grid across England and Wales. This grid size was selected as being appropriate for the spatial distribution of the sampling points (mean separation 2.5 km), and with regard to the geographical scale of LSOAs (the ultimate target geography, small areas with mean area of approximately 4 km^2^). Areal interpolation was again used to estimate an area weighted mean relative indicator of landscape quality, based on local surface water quality, for each LSOA.

### Protected and designated areas indicator

Granting protected or designated status to specific areas is a key management process for environmental protection and enhancement, with local, national and international significance [54]. The inclusion of this indicator reflects our assumption that ecological quality in protected/designated areas (PDAs) is necessarily high, an approach used in previous research [55]. Geographical boundaries of six key PDA types for Britain were obtained from the national agencies (Natural England, Countryside Council for Wales (now Natural Resources Wales), and Scottish Natural Heritage). The included area types were those primarily with ecological/biological importance: Sites of Special Scientific Interest, Special Areas of Conservation, Special Protection Areas, Local Nature Reserves, National Nature Reserves and Ramsar designated wetlands [56]. Other PDA types, such as National Parks and Areas of Outstanding Natural Beauty, were considered, but rejected due to designation on the basis of a diversity of characteristics such as cultural heritage value, aesthetics and so on. Since these areas are discrete polygons, we implemented a process that would allow for both the presence/absence of these areas, along with a certain degree of distance decay, i.e. proximity of populations to PDAs. The PDA polygons were converted to raster grids with a fine spatial resolution (25 m), and these were subsequently converted to point data (with one point per 25 m grid cell within the protected/designated area boundary). Kernel Density Estimation was carried out to estimate the relative density/proximity of these areas across Britain. This was again carried out using a 1 km grid size, and using two search radii, 10 km and 20 km. The resulting relative density measure was allocated from the 1 km grid to LSOA/DZ boundaries using an area weighted mean.

### Analysis

**Figure 1:**
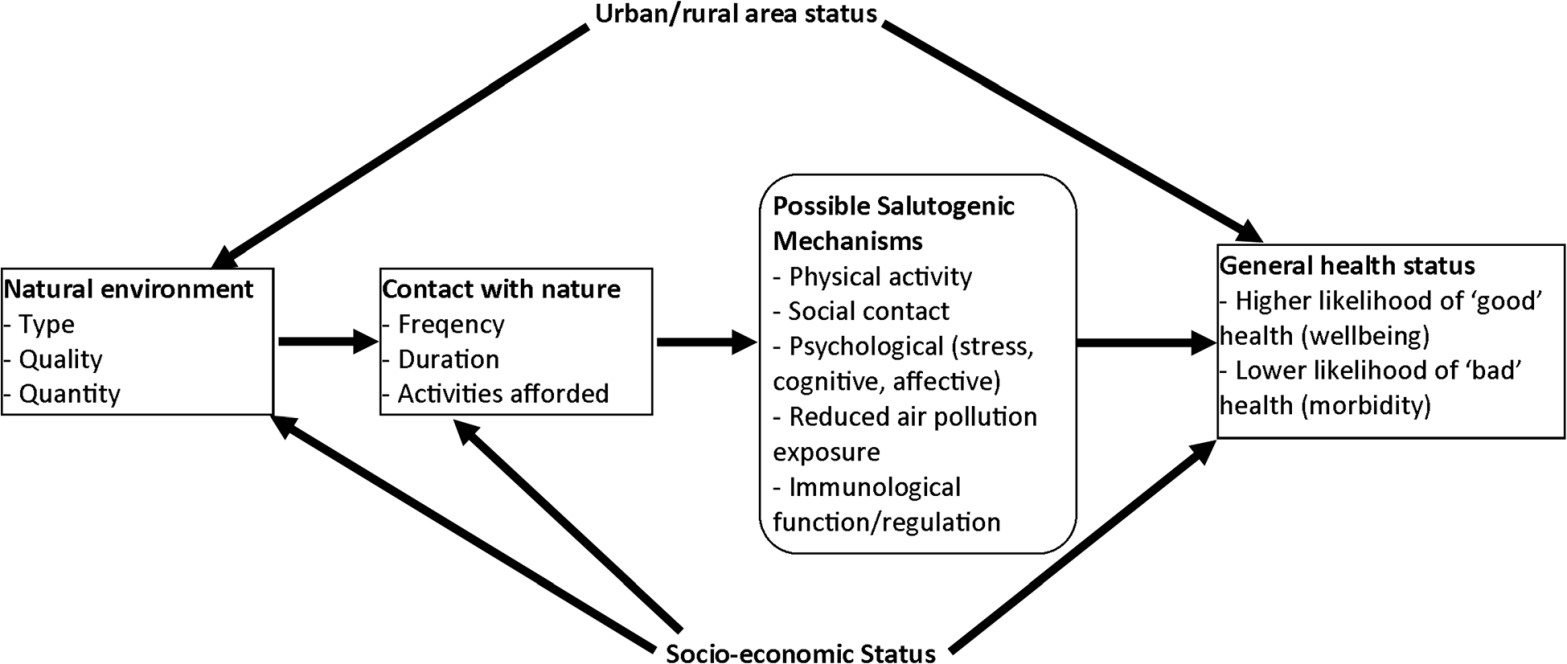
Conceptual model outlining hypothesised pathways between natural environmental type and quality and general health. Area socio-economic and urban/rural status are considered both as potential confounders and effect modifiers. Adapted from Hartig et al. (2014) [18].

A conceptual model was sketched to indicate hypothesised pathways between natural environment type and quality and general health based on one proposed previously [18], indicating potential confounders and effect modifiers (see Figure 1). The outcome measures were the LSOA age/sex standardised percentage of people with a) good/very good health and b) bad/very bad health. Ordinary least squares regression models were fit in order to estimate associations between the outcome measures, with environmental type and quality indicators as predictors. Regression analyses adjusted for income, education and employment scores of the 2010 English Indices of Deprivation as area indicators of population socio-economic status [57]. Models also adjusted for urban/rural status using a standard government classification, which classifies urban areas as ‘physical settlements’ with population of 10,000 or more [58]. Ambient air quality was initially considered for inclusion in models, but given its hypothetical role as one of the mechanisms underlying the relationship between natural environments and general health (see Figure 1) it was not modelled as a potential confounder. Due to the availability of relevant census cross-tabulations at the time of analysis, and the most recent deprivation indices (2010, based on 2001 LSOA boundaries), analyses were limited to English LSOAs remaining unchanged between 2001 and 2011 (deprivation indices were unavailable for new LSOAs).

Environment type models were built in sequence, predicting each of good and bad health prevalence by:

- Model 1. Individual land cover type proportions (each entered in separate models)
- Model 2. Model 1 + deprivation indices component scores (linear) + urban/rural status (categorical, urban as reference category)
- Model 3. Model 2 + mutual adjustment for all other natural land cover types (excluding urban)

In model 3, the proportion of land cover that is built environment is excluded from models. The interpretation for the coefficient for each land cover type is therefore the change in the health outcome associated with a percentage point increase in the local area share of that land cover, whilst the share of built environment land cover decreases equally. This approach has been used in previous work on the prediction of house prices by local land cover mix in hedonic regression modelling [59]. Similar modelling procedures were adopted for each environmental quality indicator in turn, with crude models followed by adjustment for area deprivation and urban/rural status.

Since studies suggest that effects of greenspace on health may vary by rural/urban status [7,8], and socio-economic deprivation [6], we tested for interaction between these area characteristics and environmental indicators using likelihood ratio tests to compare full models with and without interaction terms. Where effect modification was indicated, we carried out stratified analyses to explore patterns of variation in regression coefficients.

All analyses were conducted using ArcGIS 10.1 (ESRI, Redlands, USA), Geospatial Modelling Environment 0.7 (Spatial Ecology LLC) and Stata 13 (StataCorp, College Station, USA).

## Results and discussion

**Figure 2:**
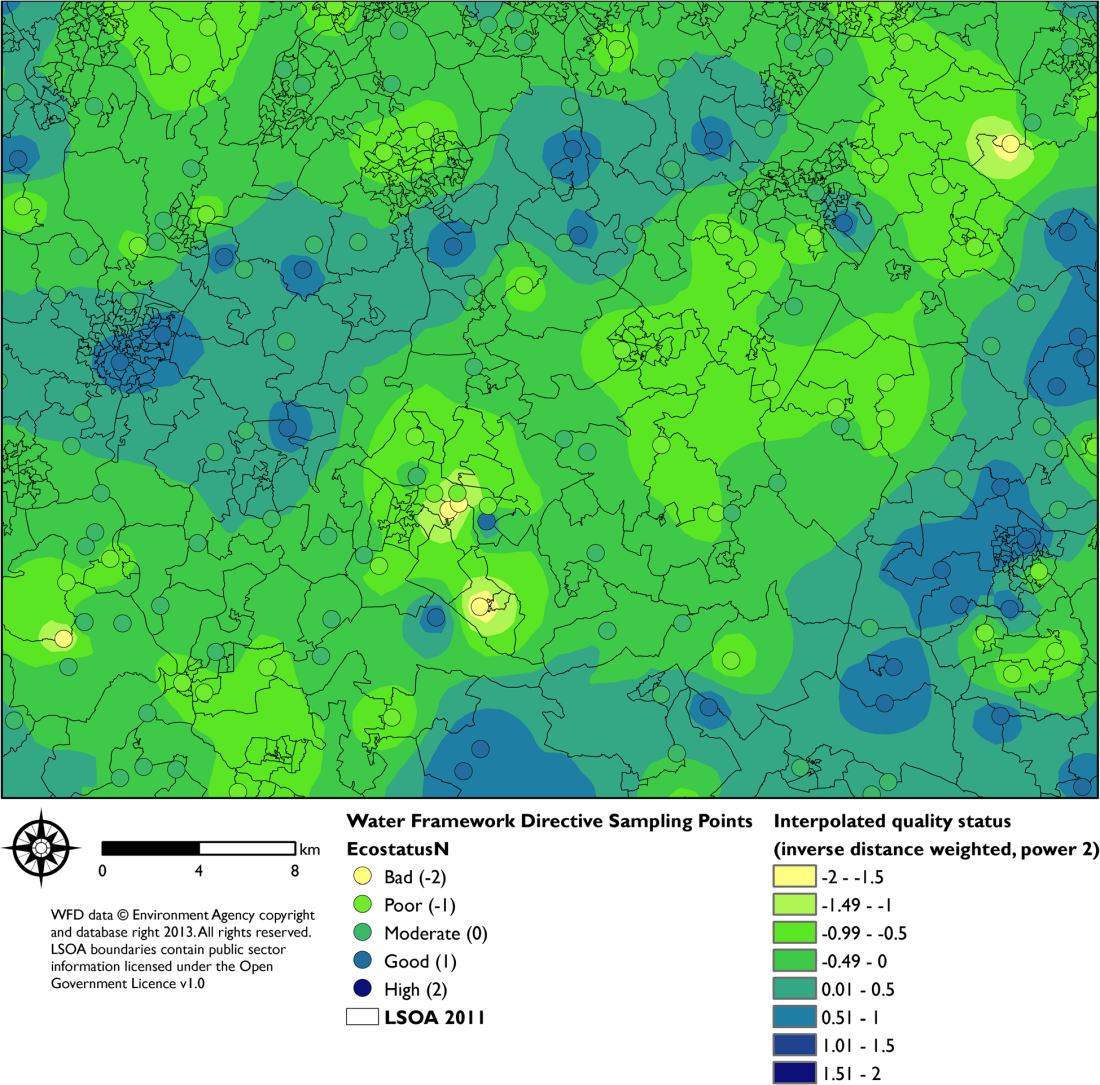
Illustration of Water Framework Directive surface water quality sampling data, and resulting interpolated indicator surface.

Table 1 presents descriptive statistics for the environmental indicators across the included LSOAs. Restricting the analytical dataset to those areas with relevant census and deprivation index data resulted in comprehensive data for 31,672 of 32,844 English LSOAs, covering 96.4% of the English population. As an example, Figure 2 depicts the source data for one of the environmental quality indicators, freshwater ecological quality, highlighting how the quality measure at each water sampling point was interpolated, with the resulting raster surface subsequently allocated to LSOA boundaries.

**Table 1:** **Descriptive statistics for environmental indicators, general health and area characteristics, for English Lower-layer Super Output Areas included in analyses (2011, n = 31,672)**

**Variable**	**Mean**	**SD** ^**e**^	**IQR** ^**e**^	**Min**	**Max**
**Landcover types (% LSOA area coverage)** ^**a**^					
Broadleaf woodland	4.37	-	5.81	0.00	88.94
Coniferous woodland	0.56	-	0.00	0.00	63.97
Arable/horticultural	13.59	-	19.93	0.00	95.53
Improved grassland	15.98	-	22.28	0.00	85.79
Semi-natural grassland	2.41	-	3.02	0.00	69.02
Mountain, heath & bog	0.67	-	0.00	0.00	72.65
Saltwater	0.03	-	0.00	0.00	38.49
Freshwater	0.49	-	0.00	0.00	77.65
Coastal	0.31	-	0.00	0.00	91.59
Urban/suburban	61.59	-	61.68	0.11	100.00
**Environmental quality indicators**					
Landcover Shannon Diversity Index (2007)^b^	0.93	0.44	0.65	0.00	2.37
Bird species richness 2008 (number of species)	87.77	10.79	14	36	141
Freshwater ecological quality indicator (2011)^c^	-0.10	0.39	0.43	-1.97	0.99
Protected/designated areas kernel density indicator (10 km search radius)^d^	81.40	-	63.42	0.00	1220.50
Protected/designated areas kernel density indicator (20 km search radius)^d^	97.50	-	78.09	1.00	1007.71
**General health (census 2011)**					
Prevalence good/very good health (Directly age/sex standardised %)	80.84	5.72	8.29	59.42	94.61
Prevalence bad/very bad health (Directly age/sex standardised %)	5.79	2.81	3.80	0.74	18.75
**Area characteristics**					
LSOA area (km2)	4.04	-	1.03	0.02	683.78
Urban	n = 26,186				
Town/fringe	n = 2,986				
Rural	n = 2,500				

^a^In each case, descriptive data are presented for each land cover type across all 31,672 LSOAs e.g. mean broadleaf woodland coverage per LSOA is 4.37% by area.

^b.^A score of 0 on the Shannon Diversity Index indicates that an LSOA is 100% covered by a single land cover type; as the number of different land covers and their relative abundance increases within the LSOA, so does the Index (e.g. an LSOA 60% urban and 40% broadleaf has a higher index than one 90% urban, 10% broadleaf).

^c^The freshwater quality indicator is interpolated from sample sites where overall ecological quality is scored from -2 (Bad) to 2 (High).

^d^The protected/designated area density indicators are relative measures with no meaningful unit; derivation is described in the text.

^e^SD = Standard Deviation; IQR = Inter-Quartile Range. Where distribution is substantially asymmetric the SD is not stated.

### Key findings: environment type & general health

Results from land cover regression models are presented in Table 2 (good health) and 3 (bad health). Coefficients represent the change in the directly age/sex standardised good or bad health prevalence (%) associated with a percentage point increase in land cover share of the relevant environment type.

**Table 2:** **Associations between land cover type density and directly age/sex standardised good/very good health prevalence (%)**

**Land Cover Map 2007 Aggregate Class**	**Model 1: Single land cover types, unadjusted**			**Model 2: Single land cover types, adjusted for IoD and urbanity**			**Model 3: Mutually adjusted for all land covers, with IoD and urbanity**		
**LSOA % land cover**	**B**	**95% CI**	**p**	**B**	**95% CI**	**p**	**B**	**95% CI**	**p**
Broadleaf woodland	**0.184**	0.176,0.193	<0.001	**0.036**	0.033,0.039	<0.001	**0.032**	0.029,0.035	<0.001
Coniferous woodland	**0.221**	0.196,0.247	<0.001	**0.017**	0.008,0.027	<0.001	-0.004	-0.013,0.006	0.449
Arable and horticulture	**0.070**	0.067,0.073	<0.001	**0.002**	0.001,0.003	0.002	**0.004**	0.002,0.005	<0.001
Improved grassland	**0.093**	0.089,0.097	<0.001	**0.018**	0.016,0.019	<0.001	**0.016**	0.014,0.018	<0.001
Semi-natural grassland	**0.186**	0.173,0.198	<0.001	**0.016**	0.011,0.020	<0.001	-0.002	-0.007,0.004	0.556
Mountain, heath, bog	**0.120**	0.102,0.137	<0.001	**0.008**	0.001,0.014	0.02	0.006	-0.001,0.013	0.082
Saltwater	*-0.379*	-0.499,-0.259	<0.001	**0.072**	0.029,0.115	0.001	**0.074**	0.032,0.117	0.001
Freshwater	**0.082**	0.054,0.109	<0.001	0.008	-0.002,0.018	0.103	0.000	-0.010,0.010	0.982
Coastal	0.019	-0.004,0.043	0.109	**0.014**	0.006,0.023	0.001	**0.019**	0.010,0.027	<0.001
Urban/suburban	-0.061	-0.063,-0.060	<0.001	-0.011	-0.012,-0.010	<0.001	a		

IoD: Indices of Deprivation. B = change in directly age/sex standardised prevalence (%) of good/very good health associated with one percentage point increase in LSOA land cover density. **Bold coefficient** - association in hypothesised direction, p < 0.05. *Italicised coefficient* - association in opposite direction to that hypothesised, p < 0.05.

^a^Urban/suburban excluded from fully adjusted model.

Most of the land cover type density measures exhibit associations with good and bad health in the hypothesised directions in crude models (Model 1), with higher densities of each natural environment (‘greenspace’) type associated with higher prevalence of good health, and lower prevalence of bad health. These associations generally attenuate to some extent following adjustment for deprivation indices and urban/rural category (Model 2), and then a small selection of ‘greenspace’ types demonstrate independent associations with health after mutual adjustment for all land cover types (Model 3). One notable exception to this pattern is saltwater coverage, which is associated with poorer health in crude models, but following adjustment this reverses to the hypothesised direction for good health prevalence, and attenuates completely for bad health prevalence. The saltwater land cover type is largely formed of estuaries, many of which are adjacent to relatively socio-economically deprived industrial/post-industrial areas. This may explain the inverse association with health in crude models, and the fact that a positive association with good health is observed in the full model is suggestive of a coastal/blue space health benefit in these areas that is only apparent after adjustment for deprivation [11]. A similar exception to the general pattern of results is coastal land cover, which does not demonstrate any association with good or bad health in crude models, but after adjustment is associated with both good and bad health in accord with the conceptual model (Figure 1), and with previous work using a coastal proximity measure [5,11].

In the full model, positive associations are indicated between good health and density of ‘broadleaf woodland’, ‘arable and horticulture’, ‘improved grassland’, ‘saltwater’ and ‘coastal environment’ types. Inverse associations with bad health prevalence are observed for the same environment types with the exception of saltwater as described above. The identification of these specific environment types is to some extent consistent with existing evidence, particularly that highlighting the potential benefits of woodlands and coasts. Whilst this may be in part due to these environment types being studied under focussed research agendas [60,61], research that has considered the spectrum of land cover types has produced comparable findings. A study using a mobile app (‘Mappiness’) to elicit georeferenced, immediate subjective assessments of happiness identified these land cover types as having amongst the strongest associations [45]. Similarly, research on visits to natural environments (including urban parks) indicated visits to woodlands and coasts amongst those with the largest association with psychological restoration [30]. It should be noted that these studies were based on specific visits to these environments, whereas the present study is based on exposure according to residential location. However, it seems reasonable to presume residential proximity relates to more frequent visits; data for England for 2012/13 indicated that 66% of visits to ‘nature’ were within 2 miles/3.2 km of home [62], and coastal residents have been shown to be 15 times more likely to have visited the coast in the week prior to interview than those living more than 20 km inland [63].

**Figure 3:**
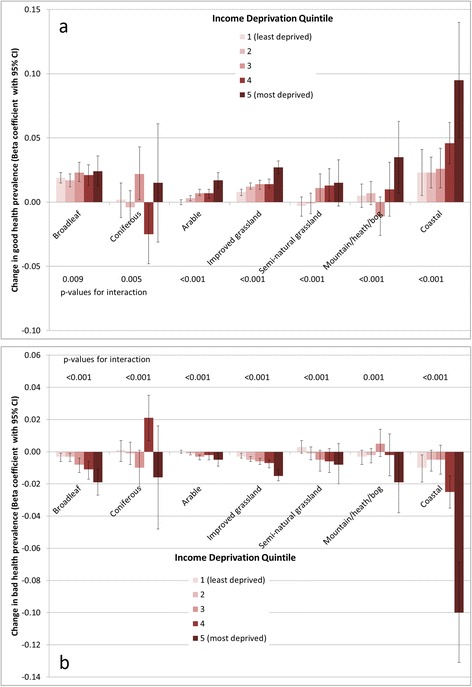
Strength of association between environment type and **a)** good health prevalence, **b)** bad health prevalence, stratified by income deprivation quintile. p-value for interaction derives from a likelihood ratio test comparing full models with and without interaction terms between the relevant land cover type and income deprivation quintile in each case.

**Figure 4:**
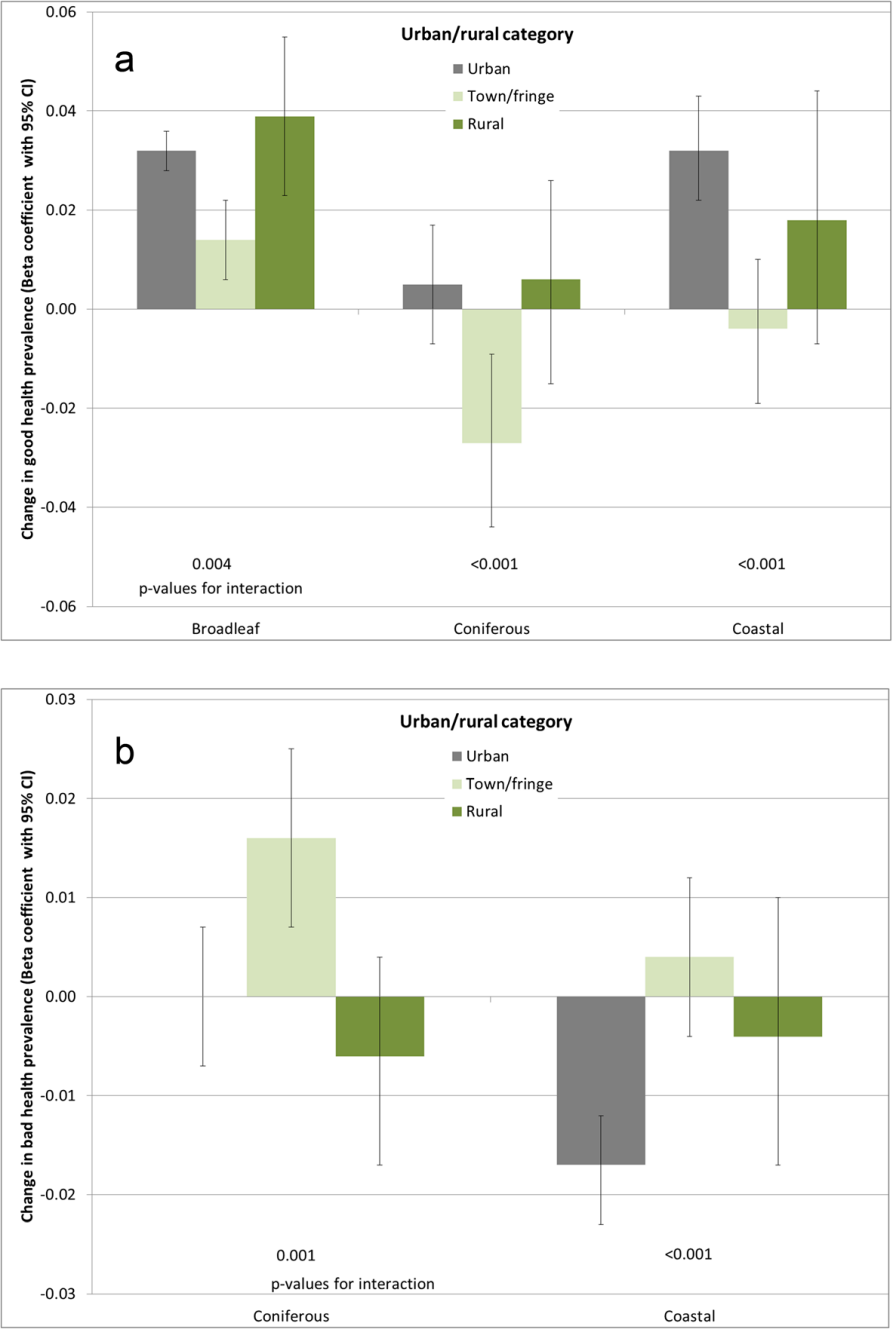
Strength of association between environment type and **a)** good health prevalence, **b)** bad health prevalence, stratified by urban/rural category. p-value for interaction derives from a likelihood ratio test comparing full models with and without interaction terms between the relevant land cover type and urban/rural category in each case.

Investigation of effect modification in associations with good and bad health indicated interaction between the density of seven land cover types with income deprivation, and three land cover types (two for bad health) with urban/rural status. Charts in Figures 3 and 4 depict regression coefficients resulting from full models stratified on income deprivation quintile and urban/rural as appropriate. Models stratified by income deprivation are mostly consistent with previous greenspace research [6,27], finding that associations are strongest in the most deprived areas, although the gradients across income deprivation quintiles do appear to vary. For example, the coefficient for coastal land cover is substantially larger in the highest deprivation quintile than in others, for both good and bad health (Figure 3), while there is a gentle gradient with regard to broadleaf woodland. Similarly, there appears to be a beneficial association between health and mountain/heath/bog land cover only in the most deprived quintile, an association which is to some extent masked when considered without stratification (Tables 2 and 3). The variation in associations with coniferous land cover across deprivation quintiles presents an unclear picture, which is difficult to interpret, but appears to be adverse in some cases. This could suggest that the health (dis)benefits of this environment type might vary according to place or specific context.

**Table 3:** **Regression results: association between land cover type density and directly age/sex standardised bad/very bad health prevalence (%)**

**Land Cover Map 2007 Aggregate Class**	**Model 1: Single land cover types, unadjusted**			**Model 2: Single land cover types, adjusted for IoD and urbanity**			**Model 3: Mutually adjusted for all land covers, with IoD and urbanity**		
**LSOA % land cover**	**B**	**95% CI**	**p**	**B**	**95% CI**	**p**	**B**	**95% CI**	**p**
Broadleaf woodland	**-0.082**	-0.086,-0.078	<0.001	**-0.010**	-0.012,-0.008	<0.001	**-0.009**	-0.011,-0.007	<0.001
Coniferous woodland	**-0.100**	-0.113,-0.088	<0.001	-0.003	-0.008,0.002	0.289	0.003	-0.002,0.008	0.283
Arable and horticulture	**-0.033**	-0.035,-0.032	<0.001	-0.001	-0.001,0.000	0.098	**-0.001**	-0.002,-0.000	0.001
Improved grassland	**-0.044**	-0.046,-0.042	<0.001	**-0.006**	-0.007,-0.006	<0.001	**-0.006**	-0.007,-0.005	<0.001
Semi-natural grassland	**-0.087**	-0.093,-0.081	<0.001	**-0.004**	-0.007,-0.002	0.001	0.001	-0.001,0.004	0.328
Mountain, heath, bog	**-0.055**	-0.063,-0.046	<0.001	-0.002	-0.005,0.002	0.341	-0.002	-0.006,0.002	0.329
Saltwater	*0.221*	0.162,0.280	<0.001	-0.011	-0.034,0.013	0.361	-0.010	-0.033,0.014	0.418
Freshwater	**-0.036**	-0.049,-0.022	<0.001	0.000	-0.005,0.006	0.914	0.003	-0.003,0.008	0.322
Coastal	-0.010	-0.021,0.002	0.106	**-0.009**	-0.014,-0.005	<0.001	**-0.011**	-0.016,-0.006	<0.001
Urban/suburban	0.029	0.028,0.030	<0.001	0.004	0.003,0.004	<0.001	a		

IoD: Indices of Deprivation. B = change in directly age/sex standardised prevalence (%) of good/very good health associated with one percentage point increase in LSOA land cover density. **Bold coefficient** - association in hypothesised direction, p < 0.05. *Italicised coefficient* - association in opposite direction to that hypothesised, p < 0.05.

^a^Urban/suburban excluded from fully adjusted model.

Interaction tests indicated effect modification by urban/rural category only for woodland and coastal environment types (Figure 4). The finding of the clearest beneficial association between coastal land cover in urban areas is consistent with previous work on coastal proximity [11]. The differential urban/rural results for woodland land cover are less easily interpreted. The association between broadleaf woodland and good health appears to be positive in all three area types, but with the association in town and fringe areas considerably weaker than in more urban or more rural areas. The pattern of coefficients for coniferous woodland is again unclear, indicating an adverse association with both good and bad health, but only amongst town and fringe areas. This is similar to the mixed findings for coniferous woodland cover by deprivation quintile, and similar interpretations of why this might be could be suggested here.

### Key findings: environmental quality & general health

Given the wide range of values for the protected/designated areas relative density indicator (0-1220 for the 10 km search radius version), versions scaled by 100 (i.e. ranging 0-12.2) were used in regression models, in order to produce coefficients for a unit increase at a reasonable scale. Full models (Model 2) for the two versions of the PDA kernel density indicator produced very similar coefficients for the association with good health (B = 0.13, 95%CI 0.11, 0.15 for the 10 km version; B = 0.10, 95%CI 0.09, 0.12 for the 20 km version), suggesting limited sensitivity to the choice of search radius for the kernel density estimation. Therefore, for the sake of parsimony, analyses are reported based on the 10 km version, as a more ‘local’ indicator of density/proximity to PDAs.

Table 4 indicates results of crude and adjusted regression models estimating associations between environmental quality indicators and good/bad health prevalence. Land cover diversity demonstrates positive associations with good health and inverse associations with bad health, which are attenuated, but not entirely, after adjustment. A similar pattern of associations is observed between the PDA density indicator and both good and bad health prevalence, with associations in the hypothesised directions remaining after adjustment. Bird species richness is still associated with good health prevalence following adjustment, but the inverse association with bad health attenuates completely in Model 2. Finally, the freshwater ecological quality indicator associates with good and bad health in the opposite direction to that hypothesised, with better water quality associated with poorer population health; these associations again attenuate, but remain, after adjustment.

**Table 4:** **Regression results: association between environmental quality indicators and directly age/sex standardised good/very good, and bad/very bad health prevalence (%)**

**Environmental quality measures**	**Model 1: Unadjusted**			**Model 2: Adjusted for IoD and urbanity**		
	**B**	**95% CI**	**p**	**B**	**95% CI**	**p**
**Good/Very Good Health**						
Shannon Land Cover Diversity Index	**2.308**	2.168,2.449	<0.001	**0.274**	0.220,0.329	<0.001
Bird species richness (+10 species)	**0.456**	0.398,0.515	<0.001	**0.022**	0.001,0.043	0.043
Freshwater ecological quality indicator	*-0.629*	-0.791,-0.467	<0.001	*-0.095*	-0.153,-0.037	0.001
Protected/designated areas kernel density indicator (10 km search radius)	**0.234**	0.184,0.284	<0.001	**0.132**	0.114,0.150	<0.001
**Bad/Very Bad Health**						
Shannon Land Cover Diversity Index	**-1.093**	-1.162,-1.024	<0.001	**-0.087**	-0.117,-0.057	<0.001
Bird species richness (+10 species)	**-0.237**	-0.265,-0.208	<0.001	0.000	-0.011,0.012	0.962
Freshwater ecological quality indicator	*0.293*	0.213,0.372	<0.001	*0.039*	0.008,0.071	0.015
Protected/designated areas kernel density indicator (10 km search radius)	**-0.100**	-0.125,-0.076	<0.001	**-0.052**	-0.061,-0.042	<0.001

B = change in directly age/sex standardised prevalence (%) of good/very good health associated with unit increase in environmental quality indicators. **Bold coefficient** - association in hypothesised direction, p < 0.05. *Italicised coefficient* - association in opposite direction to that hypothesised, p < 0.05.

The findings for the indicators of land cover diversity, biodiversity and protected/designated area density are therefore consistent with our hypotheses, and some of the previous research, that beneficial health effects of exposure to higher quality natural environments [22]. However, the inverse association between water quality and health is unexpected. It may be that this finding is somehow indicative of the ‘environmentalist’s paradox’ [64]. If degraded surface water quality is indicative of human activity (such as intensive agriculture) in the surrounding catchment, but that human activity is a source of social and economic benefit, then there may be population health and wellbeing gain *at the cost of* environmental degradation. This of course can only be speculation here, and it is not clear why this should be the case for this environmental quality indicator and not for the others (although they are measuring quite different facets of the local environment).

**Figure 5:**
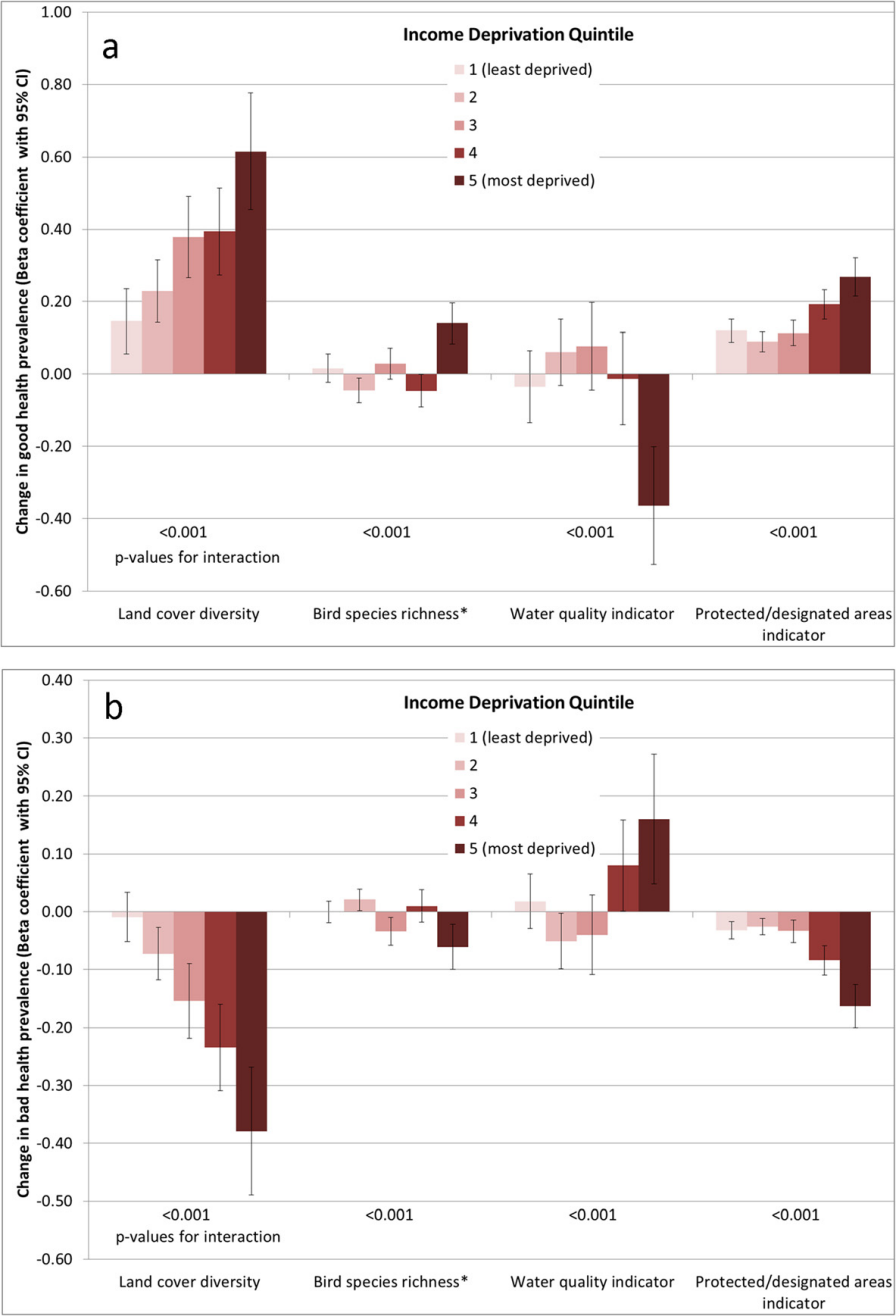
Strength of association between environmental quality indicators and **a)** good health prevalence, **b)** bad health prevalence, stratified by income deprivation quintile.*Coefficient associated with an increase of 10 species p-value for interaction derives from a likelihood ratio test comparing full models with and without interaction terms between the relevant land cover type and urban/rural category in each case.

**Figure 6:**
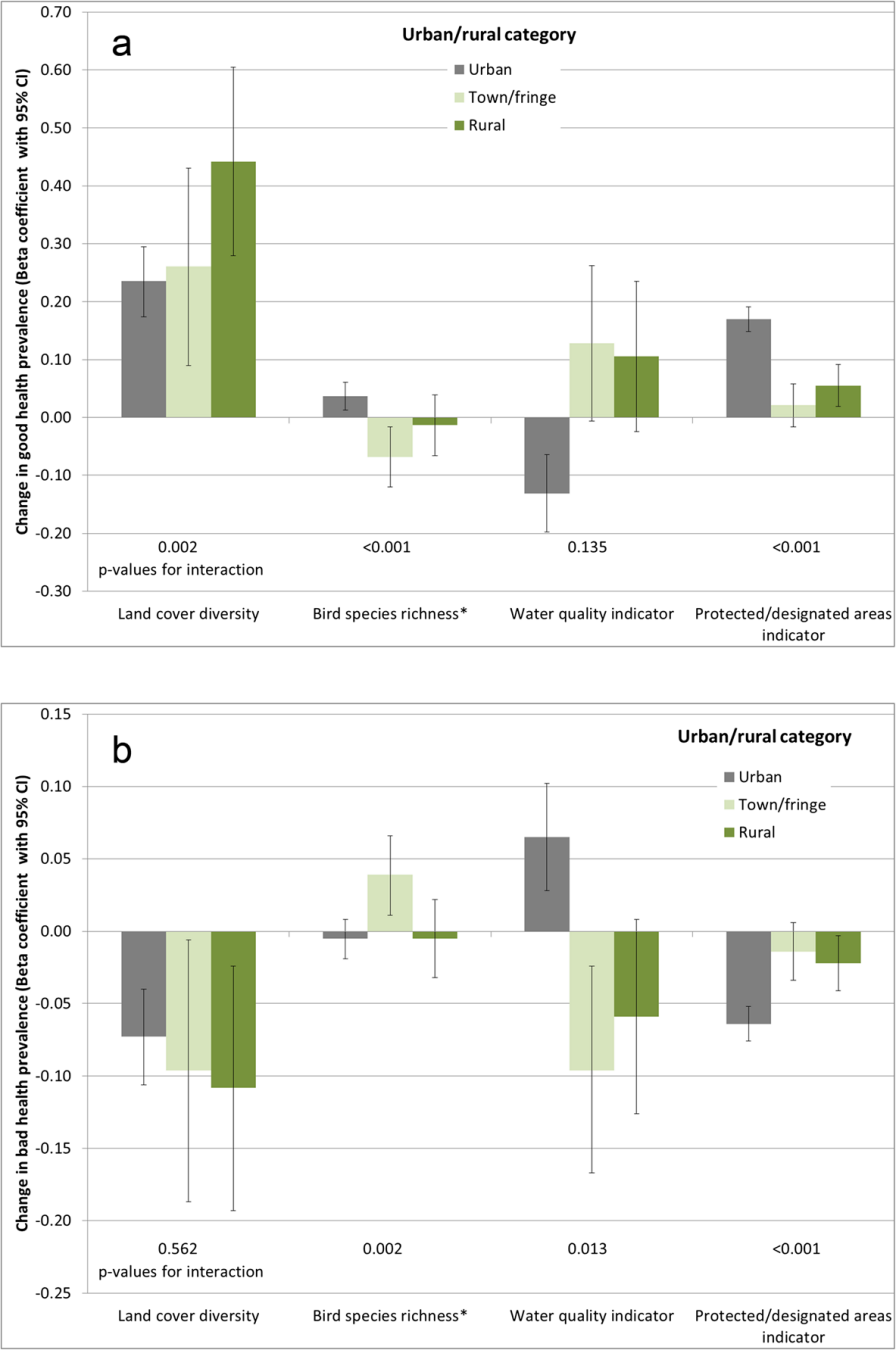
Strength of association between environmental quality indicators and **a)** good health prevalence, **b)** bad health prevalence, stratified by urban/rural category.*Coefficient associated with an increase of 10 species. p-value for interaction derives from a likelihood ratio test comparing full models with and without interaction terms between the relevant land cover type and urban/rural category in each case.

Likelihood ratio tests for good health models indicated evidence of interaction between all environmental quality indicators and income deprivation, and also with urban/rural category (with the exception of water quality, p-value for interaction = 0.135). Bad health models also suggested interactions with both income deprivation and urban/rural, with the exception of land cover diversity and urban/rural category (p = 0.562). Given these single exceptions in each case, stratified analyses were carried out for all four environmental quality indicators to investigate patterns in associations. The resulting regression coefficients are charted in Figures 5 and 6, although it should be noted that where the associated p-value for interaction is large, there is limited statistical evidence of a difference between strata. When stratified by deprivation quintile, coefficients for the land cover diversity and PDA indicators are consistent with previous evidence, suggesting stronger beneficial associations for the most deprived populations. Bird species richness demonstrates less clear gradients across deprivation quintiles, but does again suggest the strongest beneficial associations in the most deprived quintile of LSOAs. The converse appears to be the case for the water quality indicator, with the strongest adverse associations in the most deprived quintile (although without a clear deprivation gradient).

Urban/rural differentials are again mixed across the indicators. Associations with land cover diversity are stronger in more rural areas; for the PDA indicator the strongest associations are in urban areas. Bird species richness and water quality coefficients suggest opposite associations between urban/rural categories, for example with the birds indicator positively associated with good health in urban areas, but inversely associated in more rural areas. As with coniferous land cover, interpreting these differences meaningfully is difficult, and not necessarily appropriate with this type of study (see limitations below).

### Strengths & limitations

There is substantive merit in this type of study; by considering (almost) the entire population, at national scale, the study is able to take a broad, descriptive view, and to consider whether, for example, small-scale, short-term experimental findings are evident at population scale. This kind of large scale quantification therefore provides a useful adjunct to other types of study in this field, such as intervention studies [65], natural experiments [66], smaller scale, more detailed observational research [40], qualitative investigation [67] and longitudinal studies [4,68], and forms a useful part of the mix of methodological and disciplinary approaches needed to understand environmental influences on health at population scales [69].

The analysis capitalises on the power of large datasets to consider the relationship between natural environments and health across urban and rural environments, and to assess how these relationships vary between areas with differing socio-economic status. The national scope and the exploration of a variety of environmental indicators permit investigation of the potential population health impacts of a wide range of natural environmental conditions.

As an ecological, small-area study, this analysis has limited power to infer causal links between the natural environmental exposures and health outcomes considered. The study is subject to the limitations typical of this design, including inability to determine temporal sequence of exposure and outcome. Another key facet of this study design is the potential role of the ecological fallacy – we cannot presume that associations observed at this aggregate level are necessarily reflections of associations at individual level. It is worth noting here that the potential measurement errors, even if non-differential, do have the potential to bias measures of association (regression coefficients) away from the null in this type of study [70]. Additional bias may arise through the lack of age/sex standardisation of exposure variables (which is not feasible for any of those under consideration) [71].

There is also the possibility of residual confounding, especially by socio-economic status, if this is incompletely controlled for by the area measures included in models. The data are from various sources and somewhat inevitably are therefore from different time periods, although they are all within a few years of each other. The approach makes a presumption, in common with many similar studies, that small area environmental measures are a reasonable proxy for the relative exposure of local resident populations to the environmental conditions of interest [72]. Despite a mean area of only 4 km^2^, some LSOAs are considerably larger, and these areas are likely to be heterogeneous in terms of environment and population to varying extents, introducing noise into assessment of associations across all areas.

There are a number of other limitations specific to the work presented here. The environmental indicators are derived based on available, relevant data, and are not perfect representations of local environmental conditions. For example, LCM 2007 data do not include small land parcels (those <0.5 ha are dissolved into surrounding parcels). This may be of particular importance in urban areas, where ‘pocket parks’ and other small patches of non-built environment might be important features with regard to neighbourhood health and wellbeing [73]. Whilst the LCM data therefore result in some misclassification of small urban green spaces as built, comparison with a high resolution greenspace indicator used in previous research [6] with the total LCM-based non-built environment coverage gives a correlation coefficient of 0.96. In terms of the other indicators, the water quality and PDA indicators are subject to the decisions made during the processes of spatial allocation to LSOAs, and the bird species richness measure (a simple species count without regard to what would be expected for a given habitat) is a relatively simplistic indicator of biodiversity. Further, interpolated data on water quality will more accurately reflect the underlying landscape quality characteristic in areas with higher density of sampling locations. Lastly, more complex spatial modelling of environmental data could be warranted, e.g. using catchment boundaries to define the extent of water quality measurement relevance, or including consideration of proximity to different environment types, rather than being confined by statistical area boundaries.

It may also be that case, especially if psychological mechanisms are at play (see Figure 1), that perceptions or beliefs regarding environmental conditions may be more important than what is objectively measured, as has been found in some previous research [40]. The accessibility ofthe natural environment in local areas (e.g. via transport networks, access points) is also not measured here, and is likely to be important.

### Implications

In general, while there is strong statistical evidence for the associations observed here, the strength of association in each case is generally fairly weak, and the ecological design means that bias toward or away from the null is possible. Most of the spatial variation in population health is explained by socio-economic status, rather than environmental quality or type (a model predicting good health prevalence with the 9 natural land cover type measures has R^2^ = 0.15; adding the three deprivation indices increases this to 0.88). However, as has been argued previously in greenspace research [4], and in more broad consideration of population health determinants [74], small effects distributed widely across a population have the potential for substantial public health impact.

Mitchell et al. propose that good access to greenspace appears to be ‘equigenic’ – “able to disrupt the usual conversion of socio-economic inequality to health inequality” [75]. Observations here of stronger beneficial associations in more socio-economically deprived areas support this contention, especially with regard to certain environments such as the coast and protected/designated areas. The findings suggest that these natural characteristics, as well as the availability of ‘greenspace’ in the local area, may be disproportionately beneficial for more deprived communities. The application of protected or designated status is an important part of environmental management and protection. This study suggests that this recognition and safeguarding of high quality and important environments may also be particularly beneficial for human health and wellbeing, especially for urban communities.

Despite uncertainties in the evidence base, the nature, health and wellbeing axis is becoming prominent in policy across Europe. In the UK, the 2011 Public Health white paper [76] indicates the value for public health of access to greenspace, and the 2012 Public Health Outcomes Framework includes an indicator of activity in outdoor environments [77]. The 2011 Natural Environment white paper [78] links new Health and Wellbeing Boards and Local Nature Partnerships, and considers the value of nature for health. UK National Institute for Health and Clinical Care Excellence (NICE) guidance on environment and physical activity recommends that authorities “*Ensure public open spaces and public paths are maintained to a high standard. They should be safe, attractive and welcoming to everyone*” [79]. The importance of the quality of natural environments is raised in the Accessible Natural Greenspace Standard [80], but policies largely reflect the evidence, referring to greenspace in general terms. International policy also reflects the increasing consideration of linkages between environmental type and quality and human health and wellbeing. At the European level, strategies such as those relating to green infrastructure [81] and biodiversity [82] make reference to the wider health and wellbeing impacts of related policy and decision making. Globally, efforts have been made to recognise health and wellbeing within the Convention on Biological Diversity [83].

This study has focussed on the UK, but given the growing international body of evidence on links between nature, health and wellbeing (see Background), and the regional/global policy context, further research on this topic in different international settings would be valuable. Opportunities exist to construct similar measures of natural environment type and quality to those used here that could be linked to human health and wellbeing data. For example, international land cover datasets such as CORINE are available and have already been used for relevant research [84,85]; the Chinese government also recently released a 30 m resolution global landcover dataset [86]. Environmental quality measures could similarly be constructed, such as indicators of protected/designated areas using international systems such as the European NATURA 2000 network [87]. There are suggestions that various aspects of regional/local context may influence nature-health-wellbeing links, such as socio-cultural attitudes and norms, and climate-related promotion or limitation of outdoor activity [88-91]; in carrying out internationally comparable research, these issues would need to be taken into consideration.

The ecosystem services approach is now a critically important international framework for the appreciation and valuation of the impacts of different environments and ecological processes on human wellbeing [92,93]. Improving our understandings of the complexities of the relationships between different characteristics of natural environments, in their socio-spatial context, is an important part of this rapidly developing field of research and policy. It is clear that there is a need for a diverse and robust evidence base to inform relevant environmental and health policies and programmes. Despite the limitations of this particular study, it adds to the volume of evidence on nature and health in that context, and presents opportunities for further enquiry.

Future research could benefit from applying the type of indicators used here to enhance understandings of the complexities of how different environmental characteristics might best support population health and wellbeing. Additional work carried out under this project is investigating longitudinal analysis of panel data using the same environmental indicators, and localised, case study approaches to the same questions. Further work is also needed to develop and test more sophisticated methods of allocating environmental data to statistical boundaries, for example accounting for proximity rather than simple intersection.

## Conclusions

The findings presented here suggest that different natural environment types, and their quality-related characteristics, could well be differentially important for salutogenesis. Several key environment types and some of the quality indicators appear to be associated with good and bad population health as hypothesised. However, some of the indicators demonstrate inconsistent or inverse associations with general population health. Whilst subject to the limitations of the ecological design, the study contributes to the early stage evidence on this topic, and indicates that follow-up research is warranted. Studying ‘greenspace’ in its broad sense is still valuable, but these findings do indicate, in concordance with other recent research, that consideration of the complexities of natural environments is an important aspect of improving our understanding of how they relate to population health.

It is clear that there should be opportunities for simultaneously improving and protecting both natural environments and population health and wellbeing. The more that the complexities and inconsistencies of these interactions are properly understood, the more likely are policies and programmes to genuinely benefit people and our environments.

## Endnote

^a^LCM2007 Classes: Broadleaved woodland, coniferous woodland, arable and horticulture, improved grassland, rough grassland, neutral grassland, calcareous grassland, acid grassland, fen marsh and swamp, heather, heather grassland, bog, montane habitats, inland rock, saltwater, freshwater, supra-littoral rock, supra-littoral sediment, littoral rock, littoral sediment, saltmarsh, suburban, urban.

## Abbreviations

DZ: Data Zones
GLUD: Generalised Land Use Database
Km: Kilometre
LCM: Land Cover Map
LSOA: Lower-layer Super Output Areas
MENE: Monitor of Engagement with Natural Environment
NDVI: Normalised Difference Vegetation Index
NICE: National Institute for Health and Clinical Care Excellence
PDAs: Protected/designated areas
UK: United Kingdom
